# Supplementary material for: Yaws elimination in Ecuador: Findings of a serological survey of children in Esmeraldas province to evaluate interruption of transmission
Source: PLoS Negl Trop Dis. 2022 May 25;16(5):e0010173. doi: 10.1371/journal.pntd.0010173 (PMC9132314; doi:10.1371/journal.pntd.0010173)
Supplement: S1 Table — (DOCX) [file pntd.0010173.s001.docx]

**S1 Table. Results of screening tests for antibodies to *T. pallidum* antigens in 4,432 schoolchildren aged 2 to 15 years living in formerly endemic and non-endemic communities for yaws using OnSite Syphilis Ab Combo**

| Communities screened | Sample  (n=4,432) | Screening with OnSite  N (%) |
| --- | --- | --- |
|  |  |  |
| Formerly endemic region  All communities  Rio Santiago  Playa de Oro  Angostura  Playa Tigre/Playa Nueva, Zapote  Palma Real/Guayabal  Chanuzal/Pailon/Picadero  Selva Alegre  Timbire/El Porvenir  Las Antonias  Rocafuerte  Rio Cayapas  San Miguel  Mafua  Zapallo Grande  Telembi  Rio Zapallito  Boca de Zapallito  Rio Onzole  Colon  Santo Domingo | 947  40  14  45  37  31  122  121  27  31  33  15  78  74  45  131  103 | 5 (0.53%)  0  0  0  1 (2.70%)  0  2 (1.64%)  1 (0.83%)  0  0  0  0  0  0  0  1 (0.76%)  0 |
| Contiguous regions  All communities  District of Eloy Alfaro  Rio Cayapas  8 communities  Rio Santiago  3 communities  Rio Onzole  3 communities  Others  19 communities  District of San Lorenzo  14 communities  District of Quininde  10 communities | 3,485  304  340  155  1,059  1,154  473 | 48 (1.38%)  1 (0.33%)  10 (2.94%)  1 (0.65%)  18 (1.70%)  16 (1.39%)  2 (0.42%) |
