# Supplementary material for: Yaws elimination in Ecuador: Findings of a serological survey of children in Esmeraldas province to evaluate interruption of transmission
Source: PLoS Negl Trop Dis. 2022 May 25;16(5):e0010173. doi: 10.1371/journal.pntd.0010173 (PMC9132314; doi:10.1371/journal.pntd.0010173)
Supplement: S2 Table — (DOCX) [file pntd.0010173.s002.docx]

**S2 Table. Timing of sera collection by calendar year and seropositivity stratified by endemicity for yaws.**

| Year | All (n=4,432) | | | Formerly endemic  (n=947) | | | Non-endemic  (n=3,485) | | |
| --- | --- | --- | --- | --- | --- | --- | --- | --- | --- |
|  | Freq. | % | Seropos.  (%) | Freq. | % | Seropos. (%) | Freq. | % | Seropos. (%) |
| 2005 | 1025 | 23.13 | 0.88 | 675 | 71.28 | 0.59 | 350 | 10.04 | 1.43 |
| 2006 | 1490 | 33.62 | 0.34 | 119 | 12.57 | 0 | 1371 | 39.34 | 0.36 |
| 2007 | 795 | 17.94 | 0.38 | 32 | 3.38 | 0 | 763 | 21.89 | 0.39 |
| 2008 | 266 | 6.00 | 0 | 42 | 4.44 | 0 | 224 | 6.43 | 0 |
| 2009 | 106 | 2.39 | 0.94 | 79 | 8.34 | 1.27 | 27 | 0.77 | 0 |
| 2014 | 164 | 3.70 | 0 |  |  |  | 164 | 4.71 | 0 |
| 2015 | 299 | 6.75 | 0 |  |  |  | 299 | 8.58 | 0 |
| 2016 | 149 | 3.36 | 0 |  |  |  | 149 | 4.28 | 0 |
| 2017 | 138 | 3.11 | 0 |  |  |  | 138 | 3.96 | 0 |
