# Supplementary material for: Yaws elimination in Ecuador: Findings of a serological survey of children in Esmeraldas province to evaluate interruption of transmission
Source: PLoS Negl Trop Dis. 2022 May 25;16(5):e0010173. doi: 10.1371/journal.pntd.0010173 (PMC9132314; doi:10.1371/journal.pntd.0010173)
Supplement: S3 Table — Results show serological reactivity to T. pallidum antigens (any positive) further stratified into presence (active infections) or absence (past infections) of non-treponemal antibodies. (DOCX) [file pntd.0010173.s003.docx]

**S3 Table. Serological results using the confirmatory rapid test (DPP Syphilis Screen and Confirm) in 4,432 schoolchildren aged 2 to 15 years living in formerly endemic* and non-endemic communities for yaws. Results show serological reactivity to *T. pallidum* antigens (any positive) further stratified into presence (active infections) or absence (past infections) of non-treponemal antibodies.**

| Communities screened | Sample | Sex  (Male/female)  % | Median age (range)  yrs. | Positivity (%) | | | | | |
| --- | --- | --- | --- | --- | --- | --- | --- | --- | --- |
|  |  |  |  | Any positive for antibodies to *T. pallidum* | | Active infections | | Past infections | |
|  |  |  |  | N | % [95%CI] | N | % [95%CI] | N | % [95%CI] |
| All areas | 4,432 | 51/49 | 10 (2-15) | 18 | 0.41 [0.26-0.64] | 6 | 0.14 [0.06-0.30] | 12 | 0.27 [0.15-0.48] |
| Formerly endemic region  All communities  Rio Santiago  Playa de Oro  Angostura  Playa Tigre/Playa Nueva/Zapote  Palma Real/Guayabal  Chanuzal/Pailon/Picadero  Selva Alegre  Timbire/El Porvenir  Las Antonias  Rocafuerte  Rio Cayapas  San Miguel  Mafua  Zapallo Grande  Telembi  Rio Zapallito  Boca de Zapallito  Rio Onzole  Colon  Santo Domingo | 947  40  14  45  37  31  122  121  27  31  33  15  78  74  45  131  103 | 54/46  58/42  64/36  51/49  65/35  45/55  51/49  52/48  56/44  42/58  49/51  53/47  55/45  57/43  60/40  52/48  61/39 | 11 (5-15)  10 (7-15)  10 (5-14)  9 (6-15)  11 (7-15)  11 (7-15)  11 (7-15)  10 (7-15)  9 (7-15)  10 (6-13)  11 (7-15)  9 (7-15)  12 (7-15)  12 (7-15)  10 (7-15)  11 (7-15)  11 (7-15) | 5  0  0  1  1  0  2  0  0  0  0  0  0  0  0  1  0 | 0.53 [0.22-1.26]  0  0  2.22 [0.31-14.17]  2.70 [0.38-16.85]  0  1.64 [0.41-6.32]  0  0  0  0  0  0  0  0  0.76 [0.11-5.22]  0 | 1  0  0  0  0  0  1  0  0  0  0  0  0  0  0  0  0 | 0.11 [0.01-0.75]  0  0  0  0  0  0.82 [0.11-5.59]  0  0  0  0  0  0  0  0  0  0 | 4  0  0  1  1  0  1  0  0  0  0  0  0  0  0  1  0 | 0.42 [0.16-1.12]  0  0  2.22 [0.31-14.17]  2.70 [0.38-16.85]  0  0.82 [0.11-5.59]  0  0  0  0  0  0  0  0  0.76 [0.11-5.22]  0 |
| Contiguous regions  All communities  District of Eloy Alfaro  Rio Cayapas  8 communities  Rio Santiago  3 communities  Rio Onzole  3 communities  Others  19 communities  District of San Lorenzo  16 communities  District of Quininde  10 communities | 3,485  304  340  155  1,059  1,154  473 | 50/50  54/46  53/47  48/52  51/49  47/53  49/51 | 10 (2-15)  10 (6-15)  11 (6-15)  10 (6-15)  11 (6-15)  10 (2-15)  8 (8-13) | 13  0  5  0  6  2  0 | 0.37 [0.22-0.64]  0  1.47 [0.61-3.48]  0  0.57 [0.25-1.26]  0.17 [0.04-0.69]  0 | 5  0  1  0  4  0  0 | 0.14 [0.06-0.34]  0  0.29 [0.04-2.06]  0  0.38 [0.14-1.00]  0  0 | 8  0  4  0  2  2  0 | 0.23 [0.11-0.46]  0  1.18 [0.44-3.09]  0  0.19 [0.05-0.75]  0.17 [0.04-0.69]  0 |

*Endemicity defined by presence of active yaws lesions in 1988 survey [2].
