# Supplementary material for: Yaws elimination in Ecuador: Findings of a serological survey of children in Esmeraldas province to evaluate interruption of transmission
Source: PLoS Negl Trop Dis. 2022 May 25;16(5):e0010173. doi: 10.1371/journal.pntd.0010173 (PMC9132314; doi:10.1371/journal.pntd.0010173)
Supplement: S4 Table — (DOCX) [file pntd.0010173.s004.docx]

**S4 Table. Characteristics of all individuals with positivity for antibodies to *T. pallidum* antigens with and without antibodies to non-treponemal antigens using the confirmatory rapid test (DPP Syphilis Screen and Confirm)**

| **Age (years)** | **Ethnicity** | **Community** | **Region** | **Sex** | **Sample year** | **Formerly endemic** | **Treponema +** | **Non-treponema +** | **Interpretation** |
| --- | --- | --- | --- | --- | --- | --- | --- | --- | --- |
| 7 | Afro-Ecuadorian | Calderon | San Lorenzo | Male | 2007 | No | 1 | 0 | Past infection |
| 10 | Afro-Ecuadorian | Canchimalero | Eloy Alfaro | Male | 2007 | No | 1 | 1 | Active infection |
| 14 | Afro-Ecuadorian | Colon del Onzole | Rio Onzole (Eloy Alfaro) | Male | 2009 | Yes | 1 | 0 | Past infection |
| 15 | Afro-Ecuadorian | Concepcion | Rio Santiago (Eloy Alfaro) | Male | 2005 | No | 1 | 0 | Past infection |
| 8 | Afro-Ecuadorian | Cuerval | Eloy Alfaro | Male | 2006 | No | 1 | 1 | Active infection |
| 9 | Afro-Ecuadorian | Maldonado | Rio Santiago (Eloy Alfaro) | Male | 2005 | No | 1 | 0 | Past infection |
| 15 | Afro-Ecuadorian | Maldonado | Rio Santiago (Eloy Alfaro) | Female | 2005 | No | 1 | 0 | Past infection |
| 12 | Afro-Ecuadorian | Maldonado | Rio Santiago (Eloy Alfaro) | Male | 2005 | No | 1 | 0 | Past infection |
| 12 | Afro-Ecuadorian | Maldonado | Rio Santiago (Eloy Alfaro) | Female | 2005 | No | 1 | 1 | Active infection |
| 15 | Afro-Ecuadorian | Palma Real | Rio Santiago (Eloy Alfaro) | Male | 2005 | Yes | 1 | 0 | Past infection |
| 15 | Afro-Ecuadorian | Rompido | Eloy Alfaro | Male | 2006 | No | 1 | 1 | Active infection |
| 12 | Afro-Ecuadorian | Santa Rosa | Eloy Alfaro | Female | 2005 | No | 1 | 0 | Past infection |
| 14 | Afro-Ecuadorian | Santa Rosa | Eloy Alfaro | Male | 2006 | No | 1 | 0 | Past infection |
| 12 | Afro-Ecuadorian | Santa Rosa | Eloy Alfaro | Male | 2006 | No | 1 | 1 | Active infection |
| 8 | Afro-Ecuadorian | Selva Alegre | Rio Santiago (Eloy Alfaro) | Male | 2005 | Yes | 1 | 0 | Past infection |
| 14 | Afro-Ecuadorian | Selva Alegre | Rio Santiago (Eloy Alfaro) | Female | 2005 | Yes | 1 | 1 | Active infection |
| 13 | Afro-Ecuadorian | Tambillo | San Lorenzo | Female | 2007 | No | 1 | 0 | Past infection |
| 9 | Afro-Ecuadorian | Zapote | Rio Santiago (Eloy Alfaro) | Male | 2005 | Yes | 1 | 0 | Past infection |
