# Supplementary figures and images for: Yaws elimination in Ecuador: Findings of a serological survey of children in Esmeraldas province to evaluate interruption of transmission
Source: PLoS Negl Trop Dis. 2022 May 25;16(5):e0010173. doi: 10.1371/journal.pntd.0010173 (PMC9132314; doi:10.1371/journal.pntd.0010173)

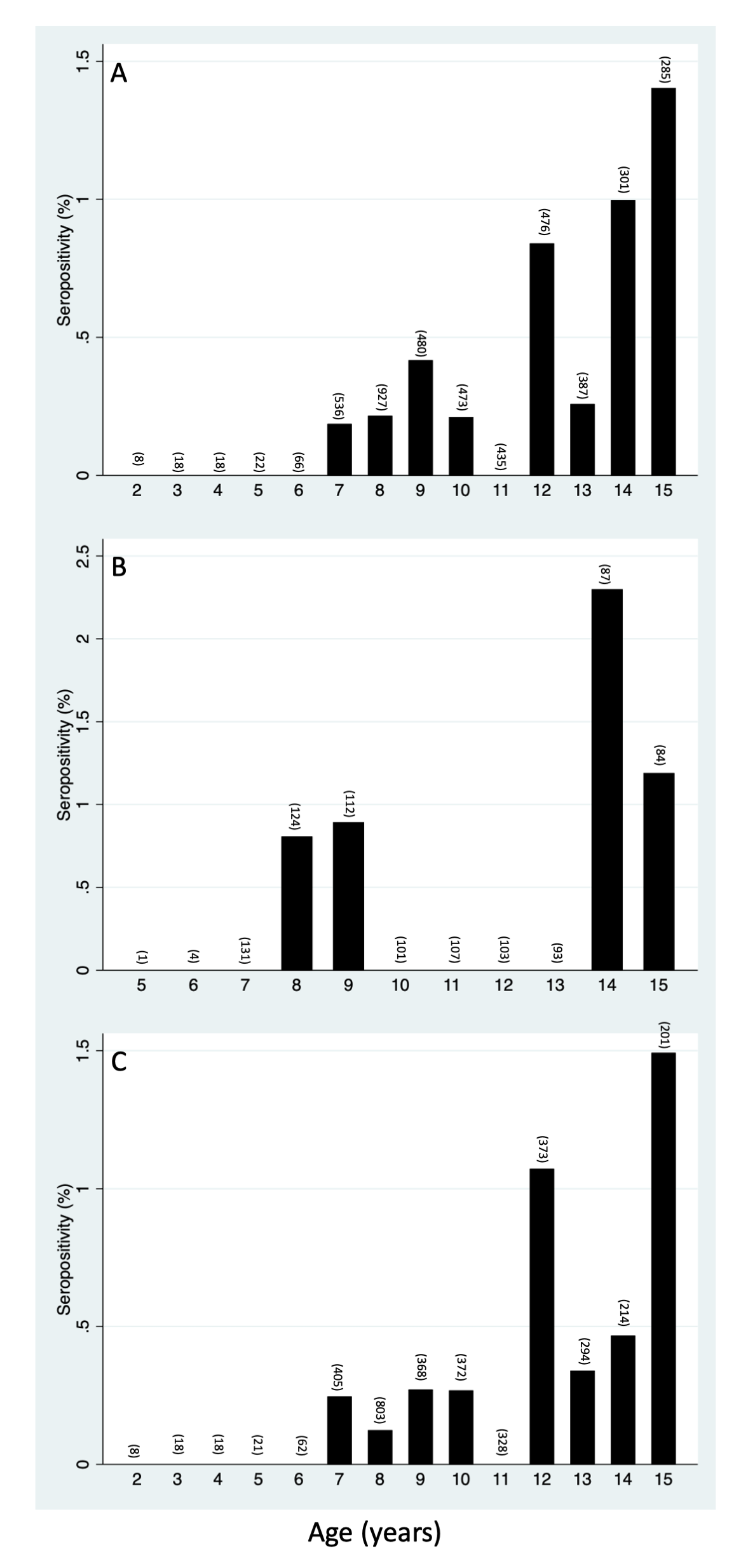

Supplement: S1 Fig — A–age-positivity in all children and stratified into children living in formerly endemic (B) and non-endemic (C) communities. Numbers in brackets represent sample numbers for each age. (TIF) [file pntd.0010173.s005.tif]
