# Supplementary material for: Yaws elimination in Ecuador: Findings of a serological survey of children in Esmeraldas province to evaluate interruption of transmission
Source: PLoS Negl Trop Dis. 2022 May 25;16(5):e0010173. doi: 10.1371/journal.pntd.0010173 (PMC9132314; doi:10.1371/journal.pntd.0010173)
Supplement: Attachment — Submitted filename: PNTD-D-22-00085_R_1_responses.docx [file pntd.0010173.s007.docx]

**Responses to reviewers’ comments**

Reviewer #1: (No Response)

Reviewer #2: The methods do not note plasma samples collected, but plasma samples are specifically mentioned in the introduction and results. Please clarify (either add plasma to methods or delete plasma elsewhere).

Response 1: “Plasma” has been deleted from the text.

Reviewer #3: The study objectives are clear with rationale explained. The study populations are described, but the  studies where samples were collected should be cited. The study design is appropriate but no sample size calculation has been included to demonstrate how the sample size was determined. This needs to be included to enable readers to see if there is adequate power behind the study. No statistical analysis plans have been presented. Only one ethics approval reference has been included despite saying these samples come from three distinct studies. All previous studies should have there ethics approval reference included.

Response 2: Studies used for the serosurvey are cited in the text (references 10-13) on page 7 of methods– specific ethical approvals for individual studies are provided in the text of each of these cited studies. The ethical approval for this retrospective analysis is provided on page 8.

Sample size was based on practical considerations based on availability of appropriate sera and number of rapid tests provided by Ecuadorian MPH/PAHO for this ad hoc retrospective analysis. The study was based on a convenience sample limited by where sampling had been done in previous non-yaws studies in formerly endemic and geographically adjacent communities. Although no positive results were expected (i.e. transmission interrupted) based on the findings of certification of yaws elimination in India (Narain et al, 2015), it was considered possible that positives might be detected because of congenital or venereal syphilis in the absence of yaws transmission. No guidance on sample size for certifying elimination of transmission is provided in current WHO guidelines for the certification of elimination of yaws (Reference 9). Guidelines for certification of elimination of another neglected tropical disease, onchocerciasis, using serological detection where expected seroprevalence is 0, recommends a sample size to detect a prevalence of <0.1% at the upper bound of the 95% confidence interval – generally a sample size of 2000 is considered to meet this criterion (WHO/CDS/CPE/CEE2001.18.b) although in practice smaller samples are acceptable given logistic and other practical considerations. It was felt that a sample of ~4,500 would provide reasonable confidence for evaluating the interruption of transmission of yaws in Ecuador’s Esmeraldas focus. We have added a new section to the Methods (page 8) on sample size and statistical analysis. Valid statistics comparing small proportions (<1%) is problematic and fails to provide robust estimates. Instead, we have compared seropositivity in formerly endemic and non-endemic communities with a population value derived from the literature using a two-tailed binomial probability test.
--------------------

Reviewer #1: (No Response)

Reviewer #2: My primary question relates to the broad time frame that the tested samples derived from. Since the positive samples were from before 2010, does that add confidence to your estimates of no/little yaws? Perhaps showing a distribution of the samples collected by year would be helpful. For example, are there no positive samples since 2010 because there are almost no samples from that time frame? Knowing that information will help us understand what the year of collection is really telling us about current yaws transmission.

Response 3: Samples collected by year and stratified by area of endemicity are shown in new supplementary table (S2 Table). The table also shows proportions of samples collected in each year and positivity rates in these samples. All samples collected from formerly endemic communities were collected over the period 2005-2009. Samples collected after 2009 (total of 750 samples representing 16.9% of samples) were from non-endemic communities. No positive samples for antibodies to *T.pallidum* antigens were observed after 2009. Explanatory text added to Results on page 11.

Reviewer #3: •  The table 1’s categories are unclear 
•       You said that there were two false positive results, I would disagree these are false positive as they were presumably positive during repeat testing using the OnSite Syphilis Ab Combo Rapid Test. Why do you believe the DPP test to be more accurate?

Response 4: Table 1 has been revised to just show the findings of sero-reactors (positive for antibodies to both *T. pallidum* and non -treponemal antigens) using the confirmatory test and the results of the screening test are now provided in S1 Table. The strategy for testing follows WHO recommendations (Reference 9). The false positives were with DPP and were interpreted as such based on being negative for antibodies to *T. pallidum* antigens but positive for antibodies for non-treponemal antigens (Castro AR et al, J Clin Microbiol 2011).

•       The lateral flow tests are looking for antibodies but you mention results being positive for antigens.

Response 5: Text has been corrected

•       “Seropositivity was observed in 0.4% of all children tested (active 0.1% and past infections 0.3%)”  this is confusing, these percentage should not be added.

Response 6: Positivity is now more explicitly defined in methods (statistical analysis) on pages 8 to 9 as follows:

“Seroreactive individuals were defined using the results of the confirmatory test as having specific antibodies to both *T. pallidum* and non-treponemal antigens. All other individuals were defined as seronegative. ….A separate analysis looked at individuals with either evidence of active (i.e seroreactors) or past/treated infections (i.e presence of antibodies only to *T. pallidum* antigens).”

•       Statistics needed, you say “Rates of seropositivity using the confirmatory assay did not differ between formerly endemic and non-endemic samples of schoolchildren (0.4% - 0.1% active infections and 0.3% past infections)” but have not presented any statistics, it is also not clear what the seropositivity rate is in each community.

Response 7: Text and Table 1 have been corrected to show seroreactivity rates with results now given to 2 decimal places. The results of the screening test are now provided in S1 table. Please see Response 2 for information on statistics.

•       I think you need to be clear throughout the paper these are not active infections now, but at the time of the sero-survey.

Response 8: Text has been clarified as requested.

•       When you say “Most communities in the formerly endemic area had no evidence of T. pallidum seropositivity but 3 communities had evidence of past infections (seropositivity ranging 0.8-4.2%) of which 1 had evidence of an active infection” what do you mean by T.pallidum seropositivity, are you distinguishing this to a past infection? I think you need to be clearer in defining terms you go on to use throughout the paper.

Response 9: Please see response 6 above.

•       Table 2 doesn’t add much that hasn’t already been described in the text

Response 10: We believe that revised Table 2 and new S4 Table provides useful summary data on seroreactives and positivity to T. pallidum antigens, respectively, relevant to the current manuscript and for future reference.

--------------------

Reviewer #1: (No Response)

Reviewer #2: Same as results - having a better understanding of the distribution of samples over the noted time frame will help us better understand the data in Table 2 - are no positive specimens since 2010 indicative of no transmission or were there not enough samples representing the time frame 2010-2017 to make any inference on recent yaws transmission.

Response 11: Please see response 3. As can be seen in the new supplementary S2 Table, no serological evidence of positivity for antibodies to *T. pallidum* antigens was detected after 2009 although samples from formerly endemic communities were all collected before this period. However, samples collected after 2009 represent only 16.9% of all samples. We have added a sentence to the results outlining this (pages 10-11).

Reviewer #3: You have concluded that the positive DPP tests found could be attributable to syphilis infection and that along with little evidence of clinical infections despite ongoing survelliance means yaws is likely to no longer be endemic. You have also mentioned how these data are important for declaring interrupted transmission and the future work that is needed to do this. Again the discussion needs proofing.

Response 12: Text of discussion has been reviewed.

--------------------

Reviewer #1: (No Response)

Reviewer #2: (No Response)

Reviewer #3: This paper should be thoroughly proof read prior to re-submission as there are many grammatical errors and lots of very long sentences. It would benefit from the use of more scientific language in parts. Make sure you correctly use and are consistent with the terms: interruption of transmission, elimination and eradication. Include missing ethics references. Make sure terms are well defined.

Response 13: The manuscript has been reviewed for errors and sentences shortened where appropriate. The following definitions have been used for interruption of transmission, elimination and eradication (Reference 9).

**Elimination of transmission (also referred to as interruption of transmission)** is defined as

“reduction to zero of the incidence of infection caused by a specific pathogen in a

defined geographic area with a minimal risk of reintroduction, as a result of deliberate

efforts; continued actions to prevent re-establishment of transmission may be required”. In the text elimination and interruption of transmission are used interchangeably.

**Eradication** of a disease is defined as the “permanent reduction to zero of the worldwide

incidence of infection caused by a specific pathogen, as a result of deliberate efforts;

with no more risk of reintroduction”.

We have corrected the text where there may be ambiguity in the use of these terms. The terms ‘elimination’ and ‘interruption of transmission’ have been used interchangeably.

--------------------

Reviewer #1: Major comments:
Methods
- Were the DPP tests read by eye or by the electronic reader?
- A map would be extremely helpful to provide a clearer understandign of the overlap between the districts and communities from which samples are available in the current study and the presumed formerly endemic districts in esmereldas. THe map provided I think (although not 100% clear from the figure legend) shows where the samples came from and if they were endemic or not endemic previously but doesnt (as far as I can tell) give a sense of what proportion of formerly endemic communities are represented in the current study.

Response 14: DPP were read by eye in a well-lit area with natural light. Text has been added to methods on page 8. The map has been revised in line with the reviewer’s comments. A sentence has been added to the Results: “Sera samples from only 3 of the formerly non-endemic communities were not available for this analysis” in page 10.

Results 
Do I read the results correctly that of 53 tests positive on the initial treponemal anibody test (and which were positive on repeat testing) 33 were negative for the treponemal component of the DPP? This is quite unusual. 

Response 15: Of the 53 (out of 4,432 sera tested) positive by the screening test, 33 were negative for antibodies to both *T. pallidum* antigen and non-treponemal antigen in the DPP test. All DPP tests were repeated twice.

Please provide at least some demographics - age, gender etc.

Response 16: Age and sex data for formerly endemic and non-endemic populations are provided now in Tables 1 and S3 Table.

Did the authors consider an age seroprevalence curve? It might be a useful supplementary figure. If there was active transmission of yaws one would anticipate a slow but consistent increase in treponemal positivity rates with age (as the test stays poisitve for life) whereas the pattern would look different in the context of venereal syphilis transmission.

Response 17: Age-positivity (individuals positive for antibodies to T. pallidum antigens) graphs are now provided as a supplementary Figure (Fig S1). These graphs show age-positivity in the whole survey (A) and stratified by endemicity (B and C). They seem to show a gradual increase in age to 15 years (1.4%) that is clearer in the non-endemic population. The number of subjects tested in the age range 2 to 6 years was likely too small (i.e. 132 individuals) to discount congenital syphilis. The increase in positivity from 7 years (graph A) could indicate active low-level transmission of yaws or sporadic cases of venereal syphilis, the risk of which increases with age. We have now added text to the Results (page 11) and to the Discussion discussing their interpretation (page 16).

There should be confidence intervals on the proportions presented throughout the manuscript.

Response 18: We have now calculated 95% confidence intervals for seropositivity that are provided in the text and Tables 1 and S3.

Minor Comments:
Some typos - for example in the introduction "Frozen sera from these studies were selected based on living a previously endemic community for yaws" is missing a word. 

Im not quite sure what is meant by the sentence:
". Only latent yaws with serological evidence of active infection can sustain transmission" do you mean treponemal antibody positivity alone doesnt find potentially infectious individuals?

Response 19: We have clarified the relevant text as follows: “Clinically active and latent (indicated here by serological evidence of active infection) yaws represent the reservoir of infection required sustain transmission.”

In the sentence "that have been shown to harbour T. pallidum pertenue-like spirochetes " Im not sure it is correct to use the word like. Phylogenetically the organism in NHPs IS t.p.pertenue.

Response 20: We have updated the reference on detection of *T. pallidum subsp. pertenue* in non-human primates. The conclusion of the authors in this study is that “Molecular data show that nonhuman primates in Tanzania are most likely infected with *T. pallidum* subsp. *pertenue*–like strains”. We have corrected then text to say “*T. pallidum* subsp. *pertenue*–like strains of spirochetes” (page 17) in line with the authors’ conclusion from a region of Africa where there were no recent reports of human yaws and thus unclear whether these non-human primate strains could infect humans.

Reviewer #2: (No Response)

Reviewer #3: This study is important for declaring elimination in Ecuador. However, there are current limitations including the lack of statistical analysis and the presentation of results as well as clear and consistent use of definitions such as seropositivity. There needs to be inclusion of a sample size calc,  statistical analysis plan and presentation of these results.

Response 21: We have added a statistical analysis section to the methods with details on sample size (see Response 2) and statistical testing (see Response 2). The term seropositivity has been more clearly defined in the text (see Response 6) and the use of definitions (i.e. elimination vs. eradication) clarified (see Response 13).
